# Springing of Plates

**Published:** 1852-10

**Authors:** J. Taylor


### *Springing of Plates.* By DR. J. TAYLOR.

Perhaps there is nothing in Mechanical Dentistry that gives more trouble than the springing (or "warping") of plates in soldering; and we know of nothing more calculated to try the patience of an operator. We presume but few in the profession can say that their patience has never been thus tried. The subject has excited a good deal of attention for the last two or three years, and we think is not still satisfactorily settled, for we still get letters asking "how can we avoid the difficulty?

We shall devote a short space to answer, as well as we can, this question. We will first try and find out a rational or philosophical cause, and then, as far as the nature of the case will permit, apply the remedy.

Some think it the unequal application of heat; some think it is owing to the alloy; some to the fact that the plate is not sufficiently confined in plaster sand and cast iron box; some think the expansion of the cast iron box helps along the difficulty, and hence use a box of sheet iron; while others use a copper ladle. Some apply heat very gradually, and others pay no attention to such precautions, and some say their plates "never warp"—"fortunate souls" they never have any trouble in any of their operations—their plugs never drop out, they never break a tooth in extracting, and they always make suction plates. But to the cause of the plate springing in the operation of soldering.

We hear a good deal of the expansive power of heat, and that metals expand in heating, and contract in cooling. These truths are too well established to bear a negative argument. Yet we wish to speak of a certain kind of contraction which takes place in the application of a high heat to gold, silver, palladium, or even platinum plate. We presume that it will be found, if a plate of either of the above metals be heated to near the fusion point, it will contract, or draw to the place at which the greatest heat is applied; or to get the idea more in full, apply heat to fusion and the plate will contract itself into a ball. This fact estalishes another, which is, that solder should flow at much less heat than is necessary to melt the plate on which it is used. We can use for soldering on platinum pure gold, and no danger of springing the plate. We can use on eighteen carat gold, alloyed with platinum, sixteen carat solder made of gold, silver and copper, and if the plate was properly prepared, should expect no springing.

Gold alloyed with copper is more elastic than when alloyed, we believe, with silver; and we attribute much of the difficulty which exists on this subject, to the combination of this metal with gold to give color, &c. to the plate. A very small quantity of iron, zinc, or lead, will make gold very brittle; hence the great uncertainty of working plate made of filings and scraps, unless these have been first thoroughly refined before drawn into plate.

It requires great care to prevent the deterioration of gold in the laboratory. The wearings from the files, the adhesion of small particles of lead in swedging, particles of zinc, &c., &c., &c., all tend to injure the working of gold. Five dwts. of scraps and filings from such gold, will render brittle and refractory fifty dwts. of pure gold. Such gold should always pass carefully through the refining process before being rolled into plate, and especially so when suction plates are required.

A few grains of zinc, of lead, or iron filings will render brittle an ounce or two of gold. Tin has the same effect, and reduces very much the amount of heat requisite to melt the gold. It is a well known fact, that solder can be made by adding a small quantity of grain tin to gold plate, which will make solder nearly as fine as the plate itself, and which will flow readily on it. This solder although beautiful in appearance, yet injures the plate, and hence has been rejected. All alloys do not effect alike the gold; an alloy of platinum gives elasticity to the gold, yet from the fact that it increases the amount of heat necessary to fuse the gold, it enables us to use a finer quality of solder without that high degree of heat which will endanger the springing of the plate. An alloy of copper gives hardness and elasticity to the gold, and as the copper contracts more than the gold in cooling, it must tend to spring the plate when used in any great amount, in either the solder or plate. We refer the reader to an article of Dr. A. C. Castle in January Number of Vol. III of Dental Register, which gives some excellent directions to prevent the springing of plates.

We regard, therefore, as the true cause of the springing of plates, the nature of the alloy, and the intensity of heat applied in soldering. In atmospheric pressure plates, we have noticed that in almost every case where the plate has been sprung in soldering, that it is raised from the palate and impinges on the outer border. In one case we punched a hole through that portion of the plate which covers the palate, and found it raised near a quarter of an inch. Many in soldering keep up a continuous jet on this portion of the plate during the operation; and often, we have no doubt, a much greater amount of heat is thrown on this part than is necessary to flow the solder.

How shall we remedy the difficulty?

We must remember that in working the plate it acquires hardness and elasticity, and in this state it is more apt to spring; heat necessarily changes this condition, and the plate is first expanded, then in cooling contracted, and if the heat is carried too far, or near the melting point, the metal tends to assume a globular form, and we have a kind of contraction on itself.

After the plate has been adapted by swedging, to the mouth, it should, as Dr. Castle remarks, be always annealed; and we would in this heat the plate as high as in soldering, for if it springs now, it will more surely spring in soldering; and for this reason—it is brought in contact with other portions of plate, united by a still baser metal, and one wnich will contract in cooling more than the plate.

A plate which has been adapted, or well fitted to the mouth, and which is changed from this adaptation by annealing, we regard as very uncertain, and prefer at once getting out new plate. Gold which is reduced in standard to eighteen carats, is not generally fit for dental purposes, and will not bear solder fine enough to resist the action of the fluid of the mouth. For atmospheric pressure plates, we prefer gold not less than twenty-one to twenty-two carats fine, and containing not more copper than is in the British sovreigns: or take pure gold and alloy with two parts silver to one of copper. Plate of such gold is more easily swedged to fit the plaster model and mouth, than of an inferior quality, and will bear the use of the finest quality of solder used in Dental Practice. Such plate, if well annealed after it is adjusted to the mouth, will not spring in soldering, unless, first by the application of too great heat; and second, by the use of too much solder containing copper, and, we may add, the union of all the backings of the teeth. We have no doubt but that this increases the diffi culty. This continuous band of gold united, and the joints filled in with solder, contracts too much for the plate in cooling; and as Dr. Castle remarks, "draws the plate upwards and inwards towards the internal median line of the central incisors. "We have tried the immersing of the plate as suggested by Dr. Castle, "in molasses," yet cannot say that any special benefit is obtained by it. It changes somewhat the color of the gold, giving it more of the copper shade. Yet we regard the thorough annealing of the plate as the great thing at this time to be accomplished. We often unite the backings on the front teeth, and then on bicuspids and molars, leaving the set in three sections.

Bad plate, with anything like good solder, will spring in spite of all external appliances; sand and plaster cannot hold it; and we have long since given up all idea of holding the plate down while soldering, by any contrivance whatever, only so far as is necessary to keep the teeth in place, and preserve them from injury by heating.

We solder in cast iron boxes, and never move our teeth after they are put in plaster and sand, until they are all soldered. We heat up our operation in a furnace, until the iron box becomes red, and then with the blow pipe pass the blaze around where we wish the solder to flow, avoiding, as much as possible, throwing a continuous jet on the center of the plate which covers the palate.

It may be asked why we use copper at all in plate or solder, if you regard it as rendering the plate more liable to spring? We answer, because we have not found it necessary to reject it altogether, and it leaves a better colored gold than silver or platinum; yet we do regard the amount usually employed as far too great.

